# An efficient vaccine against bovine leukemia virus

**DOI:** 10.1186/1742-4690-12-S1-P3

**Published:** 2015-08-28

**Authors:** Gerónimo Gutiérrez, Sabrina M Rodríguez, Agustín Vilor, Nicolas Gillet, Alix DeBrogniez, Karina Trono, Luc Willems

**Affiliations:** 1 Instituto de Virología, Centro de Investigaciones en Ciencias Veterinarias y Agronómicas, INTA, Castelar, Argentina; 2 Molecular and Cellular Epigenetics, Interdisciplinary Cluster for Applied Genoproteomics (GIGA) of University of Liège (ULg), Liège, Belgium; 3 Molecular and Cellular Biology, Gembloux Agro-Bio Tech, University of Liège (ULg), Gembloux, Belgium

## 

Previous attempts to produce a vaccine against BLV faced problems of efficacy (i.e. only a fraction of animals were protected), persistence (i.e. rapid decrease of immune protection), cost (e.g. production of purified proteins) or safety (e.g. genetically modified hybrid viruses). We have designed a novel strategy based on the use of a live-attenuated BLV provirus. The rationale behind this strategy relies on the deletion of genes required to induce pathogenesis maintaining integrity of those involved in infectivity. We have identified a BLV deleted provirus that is infectious in cattle but replicates at reduced levels in cows as shown by real-time quantitative PCR. The deletant elicits a strong anti-BLV immune response as indicated by wild-type antibody titers. Vaccinated animals but not uninfected controls resist challenge by a wild type BLV virus. The deletant does not spread to uninfected sentinels maintained during 5 years in the same herd supporting biosafety of the vaccine. Passive immunity, but not viral infection, is transmitted to the newborn calves via the maternal colostrum. Assays regarding production, storage and delivery of the vaccine, as well as safety of the milk produced by vaccinated cows are currently being carried out with the aim to start a large scale trial that will be held in Argentina in real dairy conditions during this year.

# Poster award winner - 3rd place

